# No Concordant Phylogeographies of the Rose Gall Wasp *Diplolepis rosae* (Hymenoptera, Cynipidae) and Two Associated Parasitoids across Europe

**DOI:** 10.1371/journal.pone.0047156

**Published:** 2012-10-11

**Authors:** Annette Kohnen, Iris Richter, Roland Brandl

**Affiliations:** Department of Animal Ecology, Philipps-Universität Marburg, Marburg, Germany; University Copenhagen, Denmark

## Abstract

According to the Host-tracking Hypothesis, species of higher trophic levels with a close relationship to their hosts, such as parasites or parasitoids, are expected to show spatio-temporal phylogeographic patterns similar to those of their host. Alternatively, with ecological sorting, a subset of the local species pools might shift to a related host species, thereby disengaging common phylogeographic patterns. Here, we compare the phylogeographic structures of the cynipid rose gall wasp *Diplolepis rosae* across Europe and of two of its most common parasitoids, the wasps *Orthopelma mediator* and *Glyphomerus stigma,* by analysing the sequences of two gene fragments (*COI* and *ITS 2*). The phylogeographic structures of the three species associated with roses were incongruent. *D. rosae* had the lowest genetic diversity with one major clade, *O. mediator* showed the classical phylogeographic structure for Europe with one eastern and one western clade, and *G. stigma* had the highest diversity but no geographical structuring. This discordance of geographical patterns may be explained by 1) the dispersal propensity of adult parasitoids or 2) the parasitoids having the ability to switch to another host, while the primary host becomes rare or is even not available. Furthermore there was no indication that phylogenetic patterns were affected by *Wolbachia* infections. Our results document that communities of closely interacting species may be the result of idiosyncratic biogeographic histories.

## Introduction

Plants, phytophagous insects and associated parasitoids together comprise more than 50% of the macroscopic species on Earth [1] and are therefore an important part of the world’s biodiversity. Based on the hypothesis of Ehrlich and Raven [2], ecologists long believed that co-evolution and co-speciation between plants and phytophagous insects contributed to this bewildering diversity of insects. Co-evolution requires stable communities of interacting species that may undergo parallel diversification [3]. According to the Host-tracking Hypothesis, pairs of interacting species follow the shifts of the distributional ranges of the host species with either concordance in timing (Contemporary Host-tracking Hypothesis) [4], [5] or with a temporal delay (Delayed Host-tracking Hypothesis) [6], [7]. Both forms of the hypothesis predict concordant phylogeographic structures for closely interacting species, such as symbionts, mutualists and host-parasite systems [8]–[10].

Despite these theoretical predictions the evidence for a clear concordance in the phylogeographic structure and timing of diversification of interacting species is rare [11], [12], [13]. Even related interacting species sharing the same habitat and history can differ in their genetic structure [11], [14]. Therefore, during recent years, the paradigm of interpreting the genetic structure of interacting species changed from co-evolution and co-speciation to a more individualistic view [15], [16]. Models of community assembly understand insect communities with different phylogeographic patterns as their being subsets of regional species pools that depend on the local abiotic conditions [17], [18]. One example for a community of closely interacting species are plants, gall-inducers and their associated communities. With 1300 described species, the gall wasps (Cynipidae) are one of the largest families of gall-inducing insects [19]. The induced plant galls provide nutritive tissue and a protected habitat for the gall-inducing wasp as well as for a complex community of inquilines, parasitoids and hyperparasitoids. Because of their intimate relationship and their high specialisation, a close co-distribution and co-evolution for these insect groups could be expected.

Here, we focus on the cynipid rose gall wasp system. The univoltine rose gall wasp *Diplolepis rosae* L. (Hym., Cynipidae) induces conspicuous, multi-chambered galls on *Rosa* species from several sections [20]. The galls form the basis of a complex community of parasitoids, hyperparasitoids and inquilines [21], [22]. Two of the most frequent parasitoid species are the endoparasitoid *Orthoplema mediator* Thunb. (Hym. Ichneumonidae) and the ectoparasitoid *Glyphomerus stigma* Fabr. (Hym. Torymidae) [23]. These three insect species live closely together in the same habitat, have similar life cycles, and are directly or indirectly dependent on the distribution and history of dog roses. *O. mediator* has been observed as a parasitoid of three other *Diplolepis* species, namely *D. spinosissimae* (Giraud), *D. eglanteriae* (Hartig) and *D. mayri* (Schlechtendal). These and most other *Diplolepis* species introduce much smaller galls than *D. rosae*, usually with just one chamber for one larva. Likewise, *G. stigma* attacks not only *D. rosae*, but also the inquiline species and other parasitoid species within the *D. rosae* galls. *G. stigma* has been found to attack other *Diplolepis* species in Canada, but not in Europe. Nevertheless, it is hypothesised that *D. rosae* is the main host species of both *O. mediator* and *G. stigma* [24]. Switches between different host species however would allow the parasitoid species to colonise areas where *D. rosae* is rare or even absent.

**Table 1:** Analysis of *ITS 2* and *COI* gene fragments of *Diplolepis rosae* and its most frequent parasitoids *Orthopelma mediator* and *Glyphomerus stigma.*

Wasp species	Gene fragment	Individuals (number)	Fragmentlength (bp)	Variable sites	Parsimony informative sites	Number of haplotypes	*H*	Model	Distances
*D. rosae*	*ITS* 2	28	581	4		6			
*O. mediator*		27	891	23		8	0.82	JC	
*G. stigma*		14	440	1		3			
*D. rosae*	*COI*	79	654	32	14	15	0.79	HKY+G	0.002–0.024
*O. mediator*		56	662	66	44	23	0.90	K81uf+G+I	0.002–0.062
*G. stigma*		28	609	71	33	24	0.99	HKY+G	0.003–0.040

For every wasp species and gene fragment the number of analysed individuals, the length of the fragment, the number of variable and parsimony informative sites, the number of haplotypes, the haplotype diversity (*H*), the selected evolution model with modeltest and the range of corrected distances are given.

Stille [25] suggested for *D. rosae* and another rose gall wasp species (*D. mayri*) two re-colonization routes into Sweden. One group was supposed to have come from the South and the other from Finland. Using allozymes he found for *D. rosae* very low genetic differentiation in southern Sweden [26] and higher diversity in Europe. According to his findings we expect a classical European differentiation pattern with higher diversity in the South and the differentiation of several lineages originated in glacial refugia using different re-colonization routes [27]–[31].

Furthermore the reproductive strategy of one species might be important for their current geographical differentiation because of differences in dispersal and consecutive settlement abilities [14]. Parthenogenetic reproduction could reduce gene flow between populations [32] and might therefore lead to a higher genetic differentiation between populations as well as to a lower genetic variability within populations [33]. Parthenogenetic reproduction can be induced by the intracellular bacteria *Wolbachia* [34]. The type of *Wolbachia* and the infection rate of the three chosen insect species differ. The gall inducer *D. rosae* is infected with *Wolbachia* type I, which induces parthenogenesis and has a recent infection in central Germany of more than 99% [35], [36]. In the study of Schilthuizen and Stouthamer [35], *O. mediator* was infected with *Wolbachia* type II, but only at one of three sampling sites. The reproductive impact on *O. mediator* is currently not known. Infections of *G. stigma* with *Wolbachia* have not been observed [35].

Here we tested two alternative scenarios for the biogeographic histories of *Diplolepis* and their parasitoids. First, all three insect species have co-evolved, had undergone similar population dynamics and are influenced by their associated life cycles in similar ways. Assuming a tight co-distribution of gall wasps and parasitoids in space and time the same basic patterns of genetic differentiation should be found in gall wasps as well as parasitoid species. Second, the possibility to switch between host species disengaged the close connection of the distributions of both the gall wasp and associated chalcid parasitoids [7]. Therefore the same basic patterns for all three species would not be expected. The parasitoid species may have survived or expanded in regions where *D. rosae* did not occur. This should have lead to spatial discordance in distributional patterns of genetic diversity. Therefore we would expect different phylogeographic patterns for the parasitoids and their host species. We tested these scenarios using sequences of variable regions in the DNA of the wasps and the parasitoids. One mitochondrial marker and one nuclear locus were chosen [37], [38].

**Figure 1:**
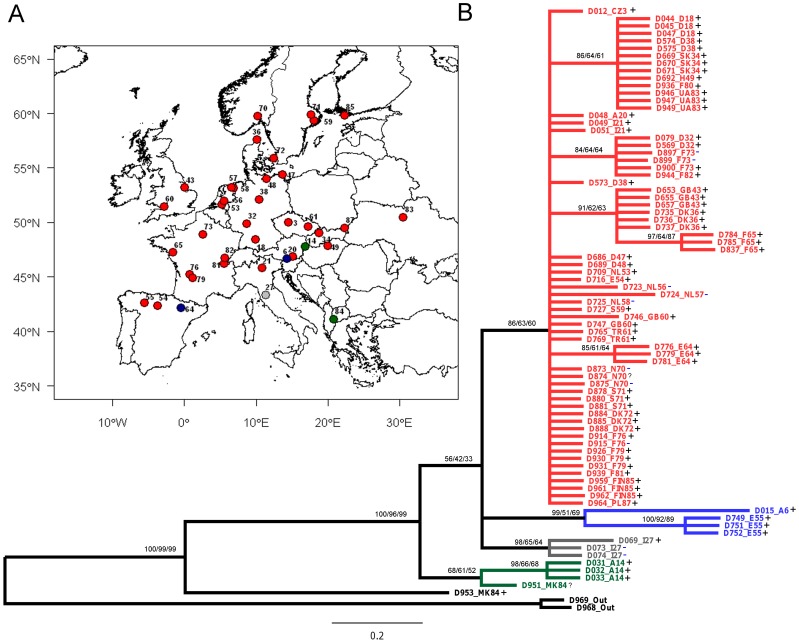
Relationship of the *COI* haplotypes of *Diplolepis rosae* in Europe. **A**. Geographical distribution of *D. rosae* samples. Colour codes correspond with clades **B**. Bayesian 50% majority-rule consensus tree for the *COI* data of *D. rosae*. The first number at each node indicates posterior probability values; the second and third numbers indicate bootstrap support of the corresponding clades of neighbour-joining and maximum likelihood trees. With the taxon labels the country and number of sampling site are given. *Wolbachia* infection is indicated with +, absence with -.

## Materials and Methods

### Sample Collection

Galls of *D. rosae* were collected in Europe during 2006 and 2007. All sampling sites (not privately owned or protected) and collectors (no specific permits were required because no protected or endangered species were involved) are named in the Table S1. Each gall was kept in separate plastic pots covered with gauze. Inhabitants of the galls were allowed to exit the galls until July and subsequently identified morphologically and stored in 90% ethanol. Only one individual of each species per gall was used for genetic analyses to avoid sampling of closely related siblings or identical samples owing to parthenogenesis in *D. rosae*.

### Molecular Methods

Total DNA of the emerged gall inhabitants was extracted using spin columns (DNeasy tissue kit, Qiagen, Hilden, Germany). We amplified and sequenced two DNA fragments: *ITS 2,* with a length of ca. 700 bp; and the mitochondrial *COI,* with a length of ca. 650 bp.

The *ITS 2* fragments of *D. rosae* and *G. stigma* were amplified with the primers forward ITS5.8F (5′-GTC CAC GGA TAC AAT TCC CGG ACC-3′) [39] and reverse ITS 4 (5′-TCC TCC GCT TAT TGA TAT GC-3′) [40]. Amplifications consisted of an initial denaturation at 95°C for 2 min, then 30 cycles with denaturation at 95°C for 30 s, annealing at 55°C for 1 min and extension at 72°C for 1 min. These steps were followed by extension at 72°C for 10 min.

The *ITS 2* fragment of *O. mediator* was amplified with the primers *ITS 2* F (5′-GGG TCG ATG AAG AAC GCA GC-3′) and *ITS 2* R (5′-ATA TGC TTA AAT TCA GCG GG-3′) [41]. Amplification consisted of 35 cycles with an annealing temperature of 51°C for 1 min.

The *COI* fragment from all three insect species was amplified with the primers forward LCO (5′-GGT CAA CAA ATC ATA AAG ATA TTG G-3′) and reverse HCO (5′-TAA ACT TCA GGG TGA CCA AAA AAT CA-3′) [42]. Amplification consisted of an initial denaturation at 95°C for 5 min, then 35 cycles with denaturation at 95°C for 30 s, annealing for 45 s at 45°C for *D. rosae* and at 40°C for *O. mediator* and *G. stigma,* and extension at 72°C for 1 min. These steps were followed by extension at 72°C for 10 min.

All reactions were carried out in 20 *µ*l reaction volume containing 2–5 *µ*l of template DNA, 10 mM Tris-HCl (pH 8.3), 50 mM KCl, 1.5 mM MgCl_2_, 80 *µ*M dNTP, 10 *µ*M of each primer and 1 unit of *Taq* DNA polymerase (New England Biolabs, Frankfurt a. Main, Germany). *ITS 2* fragments of *O. mediator* were extracted from an agarose gel using the QIAquick gel extraction kit (Qiagen, Hilden, Germany). All other PCR products were purified using a Qiagen MinElute PCR purification kit. Fragments were sequenced directly by Sequencing Laboratories Göttingen GmbH, Germany.

**Figure 2:**
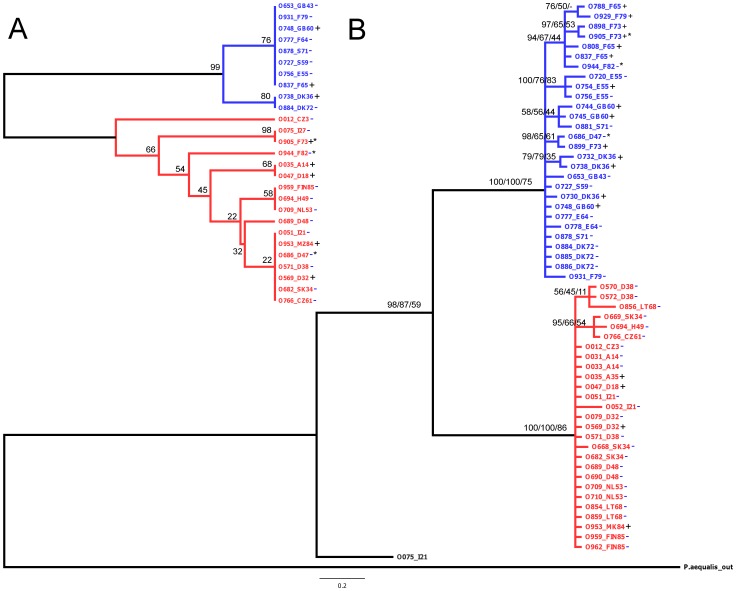
Relationship of *Orthopelma mediator* in Europe. **A**. Neighbour-joining tree of *ITS* data. Numbers at nodes indicate bootstrap support. **B**. Bayesian 50% majority-rule consensus tree for *COI* data of *O. mediator*. The first number at each node indicates posterior probability values; the second and third numbers indicate bootstrap support of the corresponding clades of neighbour-joining and maximum likelihood trees. With the taxon labels the country and number of sampling site are given, see Fig. 3. *Wolbachia* infection is indicated with +, absence with -. The western clade is coloured in blue, the eastern in red. Individuals with conflicting assignment are marked with *.

**Figure 3:**
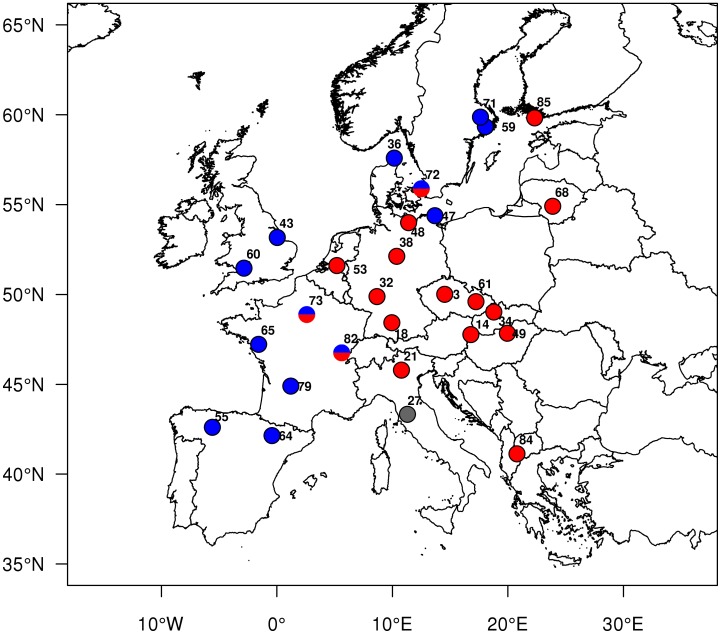
Geographical distribution of the eastern and western clades of *Orthopelma mediator* in Europe. Haplotypes were assigned based on the *COI* and *ITS* 2 sequences. Blue  =  western clade, red  =  eastern clade, grey  =  haplotype found only in Italy, half blue/half red  =  individuals assigned to eastern clade based on *ITS* 2 sequences and to the western clade based on *COI* sequences.

### Infection of Wasps by *Wolbachia sp*

The presence or absence of *Wolbachia* was tested with specific primer pairs amplifying ca. 600 bp of the *wsp* gene using primers forward *wsp* 81F (5′-TGG TCC AAT AAG TGA TGA AGA AAC-3′) and reverse *wsp* 691R (5′-AAA AAT TAA ACG CTA CTC CA-3′) [43]. Whether the absence of a PCR product was caused either by the absence of *Wolbachia* or by a reaction failure was checked with a control primer pair. Control primers for *D. rosae* were forward Dr06-F (5′-CTC ATC TCT TCT TCT TAT CTC AG-3′) and reverse Dr06-R (5′-CCC AGG AGA GCA GAG G-3′) [33], and control primers for *O. mediator* and *G. stigma* were LCO and HCO [42]. All PCR reactions had both a positive control (known infected individual) and a negative control (water). Amplifications consisted of initial denaturation at 94°C for 3 min, then 35 cycles with denaturation at 94°C for 1 min, annealing at 50°C (*D. rosae)* or 42°C (*O. mediator* and *G. stigma)* for 1 min and extension at 72°C for 1 min. These steps were followed by extension at 72°C for 5 min.

### Phylogenetic Analysis

Sequences were manually edited and aligned using BioEdit version 7.0.9 [44]. Haplotype frequencies and diversities were estimated using the program DnaSP version 4.10 [45]. The hierarchical likelihood ratio tests and the Akaike information criterion implemented in MODELTEST 3.7 [46] were used to select appropriate models of sequence evolution including outgroup individuals (for *D. rosae,* two individuals of *D. fructuum,* GenBank JN252403, JN252404; for the *COI* sequences of *O. mediator,* one individual of *Pimpla aequalis* from GenBank AF146681; and for *COI* sequences of *G. stigma,* one individual of *Nassonia vitripennis* from GenBank EU746551).

The selected models (Table 1) were implemented to calculate corrected distances and to construct phylogeographic relationships by using the neighbour-joining algorithm as well as using maximum likelihood and Bayesian methods. For the latter we used the program MrBayes version 3.1.2 [47]. Four chains per run and two independent runs were used. A run length of 1 million generations and a sample frequency of every 1000 generations were preset with a burnin period of 50000 generations. All analyses were checked for convergence with the program Tracer v1.5 [48]. For the sequences of *O. mediator* both gene fragments were analysed separately and combined. For neighbour joining and maximum-likelihood analyses (using a heuristic search algorithm) data were supplied to the programs MEGA version 4 [49] and PAUP* version 4.0b10 [50]. To quantify the reliability of the nodes, 1,000 bootstrap replicates were used for these two approaches. For *COI* sequences of *O. mediator,* we used analysis of molecular variance (AMOVA) Arlequin version 3.11 [51].

**Figure 4:**
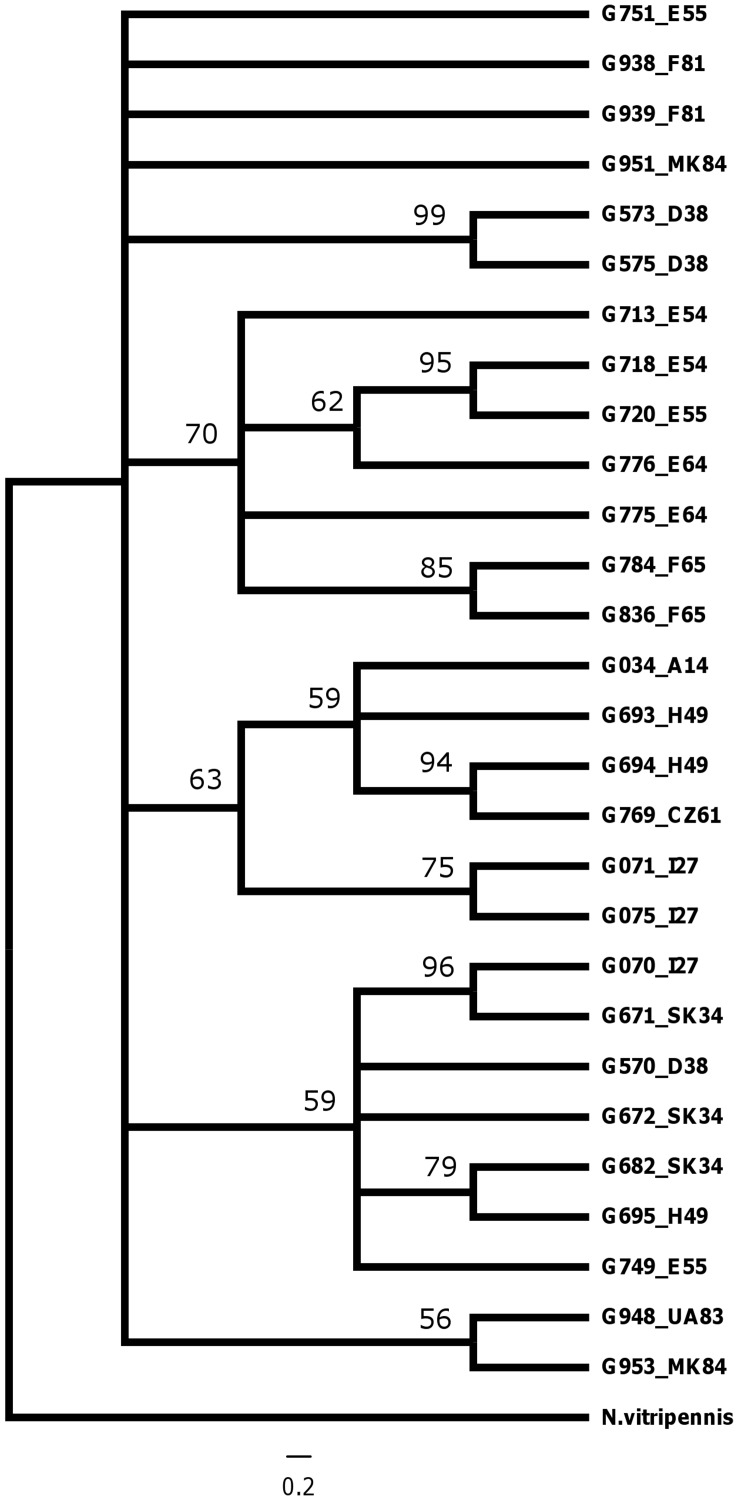
Bayesian 50% majority rule consensus tree of *COI* data of *Glyphomerus stigma*. Numbers at nodes indicate posterior probabilities. With the taxon labels the country and number of sampling site are given, see Table S1, compare with Fig. 1 and 3.

## Results

### *Diplolepis Rosae*

The *D. rosae* mtDNA sequences of *COI* (GenBank JN252324-JN252338) had many more variable sites than the genomic *ITS 2* sequences (GenBank JN252386-JN252391) (Table 1). For both loci, we found one frequent haplotype and several haplotypes that occurred only once (Table S1). Individuals from the same sampling site carried the same haplotype, with three exceptions.

For *COI* sequences, Modeltest selected the model of sequence evolution of Hasegawa, Kishino and Yano [52] with among site rate variation. In all topologies one major clade was found which was common across Central and Northern Europe and was also recorded in Spain (Fig. 1). Three subclades were recorded only in Southern Europe (Fig. 1A).

### *Orthopelma Mediator*

As for *D. rosae*, *COI* sequences of *O. mediator* (GenBank JN252339-JN252361) were much more variable than *ITS 2* sequences (GenBank JN252392-JN252399); the *ITS 2* sequences had two positions at which one or two base pairs were deleted (Table 1). The most frequent haplotype of *COI* was found in 14 individuals (n = 56). For *ITS 2,* Modeltest selected the simplest model of sequence divergence, the Jukes-Cantor model [53], and for *COI,* Modeltest selected the K81 model with unequal base frequencies [54].

All topologies with all three methods and both markers separately or combined divided *O. mediator* into two major clades (Fig. 2, Fig. S1). One clade was found in Western Europe, distributed from Spain to France, the UK, and Denmark, up to Sweden (western clade). The other clade was found in Macedonia, Central Europe, and Lithuania up to Finland (eastern clade, Fig. 3). For three individuals, the two loci showed incongruent clade affiliation (Fig. 2, 3, S1); the *ITS 2* sequences assigned all three individuals to the eastern clade, but the *COI* sequences assigned them to the western clade. In the combined analyses the three individuals were assigned to the western clade. A minor, third clade only distantly related to the two main clades and containing only one individual (from Italy) was identified by the *COI* sequences and the combined dataset (Fig. 3, S1). Both western and eastern clades were supported by high bootstrap values and showed low genetic distances within (0.45 and 0.58%, respectively) and higher distances between (5.2%) each other. An AMOVA attributed only 6% variation within clades (SS = 53, df = 53), but 94% between clades (SS = 422, df = 1). The pair-wise F_ST_ value between clades was 0.94 (p<0.0001). The haplotype diversity *H* in the eastern and western clades was 0.66 and 0.91, respectively.

### *Glyphomerus Stigma*

The *ITS 2* sequences of *G. stigma* individuals (GenBank JN252400-JN252402) were almost identical, with only one variable position in four individuals from Italy, Macedonia, Ukraine and the Czech Republic. The individual from Ukraine also had a deletion of two base pairs. In contrast, the *COI* sequences of *G. stigma* individuals (GenBank JN252362-JN252385) were highly variable (Table 1). Most haplotypes were found only once, and four haplotypes were found twice; modeltest selected the Hasegawa, Kishino and Yano model with among site rate variation [52]. In all topologies,haplotypes were clustered together with high support, but no consistent clades are supported between haplotypes (Fig. 4).

### *Wolbachia* Infection

*Wolbachia* infection was common in the gall wasp *D. rosae* (91%; n = 79, Fig. 1). We found no geographic pattern in the infection rate. The parasitoid *O. mediator* showed a much lower infection by *Wolbachia*; 10% of the sampled individuals were infected (n = 56). Populations with infected individuals were distributed throughout Europe but not in Scandinavia (Fig. 2). The parasitoid *G. stigma* (n = 28) was not infected with *Wolbachia* bacteria. The overall infection across species differed significantly (chi-square 27.6, df = 2, p<0.001).

## Discussion

The phylogeographical structure of *D. rosae* and two of its most common parasitoids, *O. mediator* and *G. stigma*, showed little phylogeographic congruence across Europe. Of the two analysed sequences just one, the mitochondrial COI fragment, was variable. The ITS 2 region showed no or little variation in *G. stigma* and *D. rosae*. Therefore our results and conclusions are mainly based on the mitochondrial fragment. Due to gene duplication, loss, lineage sorting and horizontal transfer gene trees need not necessarily reflect species trees [55], [56]. Therefore in a further study our results should be verified with data from more genes.

The most common host plants of *D. rosae* are dog roses of the section *Caninae*. Dog roses comprise more than 150 species that hybridise and differ in several characters. Little is known about their biogeographic history except that this section originated by hybridisation events presumably during the last ice ages [57]–[59]. By using almost all lineages of dog roses as a host, the gall wasp is able to colonise the entire palaearctic region. Compared to its two parasitoid species, *D. rosae* showed low haplotype diversity and low genetic distances between haplotypes. A similar low genetic diversity was found by Stille [26] with allozymes in Swedish populations. In contrast *D. mayri* shows higher diversities and a division in two subpopulations in Sweden, which were also geographically divided pointing to two different re-colonisation routes after the last ice ages [25]. The distribution of the common haplotype of *D. rosae* across Europe points to ongoing high gene flow between sampling sites [36]. The higher haplotype richness in Southern Europe is consistent with the expectation of Pleistocene refugia in that area [27]–[30] and a re-colonisation of the regions north of the Alps by the common haplotype.

In contrast to the gall wasp, the parasitoid species showed much higher haplotype diversities. We found no clear geographical structure for *G. stigma* for either locus, which points either to high rates of mixing and exchange between populations (and therefore also to good dispersal abilities) or to a large effective population size with incomplete lineage sorting of these specific genes. The pronounced divergence of *O. mediator* into two lineages, an eastern and a western clade, is a classical pattern found in many plant and animal species [27], [31]. This split is thought to be the result of survival during the Pleistocene in isolated refugia one located in Iberia and a second one somewhere in the east maybe in the Balkans. In a suture zone between both clades in France and Germany, where both clades live in sympatry, we found three “hybrid” individuals with contrasting mitochondrial and nuclear haplotypes.

Some populations of *O. mediator* of both geographical lineages were infected with *Wolbachia*. These bacteria are normally vertically transmitted from the mother to offspring with the cytoplasm of eggs. Some phylogenetic studies however have shown that phylogenies of insect species and associated bacteria are incongruent, which suggests that horizontal transmission must have taken place frequently [60], [61]. Schilthuizen and Stouthamer [35] found no horizontal transmission between *D. rosae* gall wasps and parasitoids, because host and parasitoids are infected with different types or strains of *Wolbachia*. We found no congruent geographical pattern in the *Wolbachia* infection of host and parasitoids leading to the conclusion that the infection by *Wolbachia* had little influence on the phylogeographic structure of hosts and parasitoids. In a recent study, Nicholls and colleagues [62] have found congruence of lineage divergence as well as temporal patterns within and across trophic levels of two parasitoid species of the genus *Megastigmus,* both assoociated with oak gall wasps. But this is one rare example and less than a general pattern within oak gall wasps and their parasitoids. Several oak gall wasps have shown broadly compatible geographical distributions and genetic structures with an eastern origin and a re-colonization through central Europe [6], [62]–[66], but not always on the same timescales. In more recent times oak gall wasps restricted to *Quercus* section Cerris expanded their ranges northwards because of human mediated plantings of their host trees [6], [64]. No such change in the distribution and such strict restriction to one host plant section is known for *D. rosae*.

The different phylogeographical patterns of the three interacting insect species studied here may be explained by host switching of the parasitoids when the primary host was not available. Indeed, *O. mediator* and *G.stigma* have been observed as parasitoids of other *Diplolepis* species. Since parasitoids are bound to their host species only during the larval stage, further dispersal and host switching is possible at later stages. Dispersal abilities and the ensuing colonisation success also influence population structures [14]. Dispersal and host switching thereby disengages the close association in the geographic dynamics of the gall wasp and its parasitoids.

## Supporting Information

Figure S1 **Bayesian 50% majority rule consensus tree of** ***COI*** **and** ***ITS 2 sequences combined*** **of Orthopelma mediator.** The number at each node indicates posterior probability values. With the taxon labels the country and number of sampling site are given, see Fig. 3. The western clade is coloured in blue, the eastern in red.
(TIFF)

Table S1 Sampling sites where rose galls were collected, collectors, number of individual wasps collected and their haplotype: the gall wasp Diplolepis rosae and its most frequent parasitoids Orthopelma mediator and Glyphomerus stigma.
(DOC)
