# Supplementary figures and images for: No Concordant Phylogeographies of the Rose Gall Wasp Diplolepis rosae (Hymenoptera, Cynipidae) and Two Associated Parasitoids across Europe
Source: PLoS One. 2012 Oct 11;7(10):e47156. doi: 10.1371/journal.pone.0047156 (PMC3469489; doi:10.1371/journal.pone.0047156)

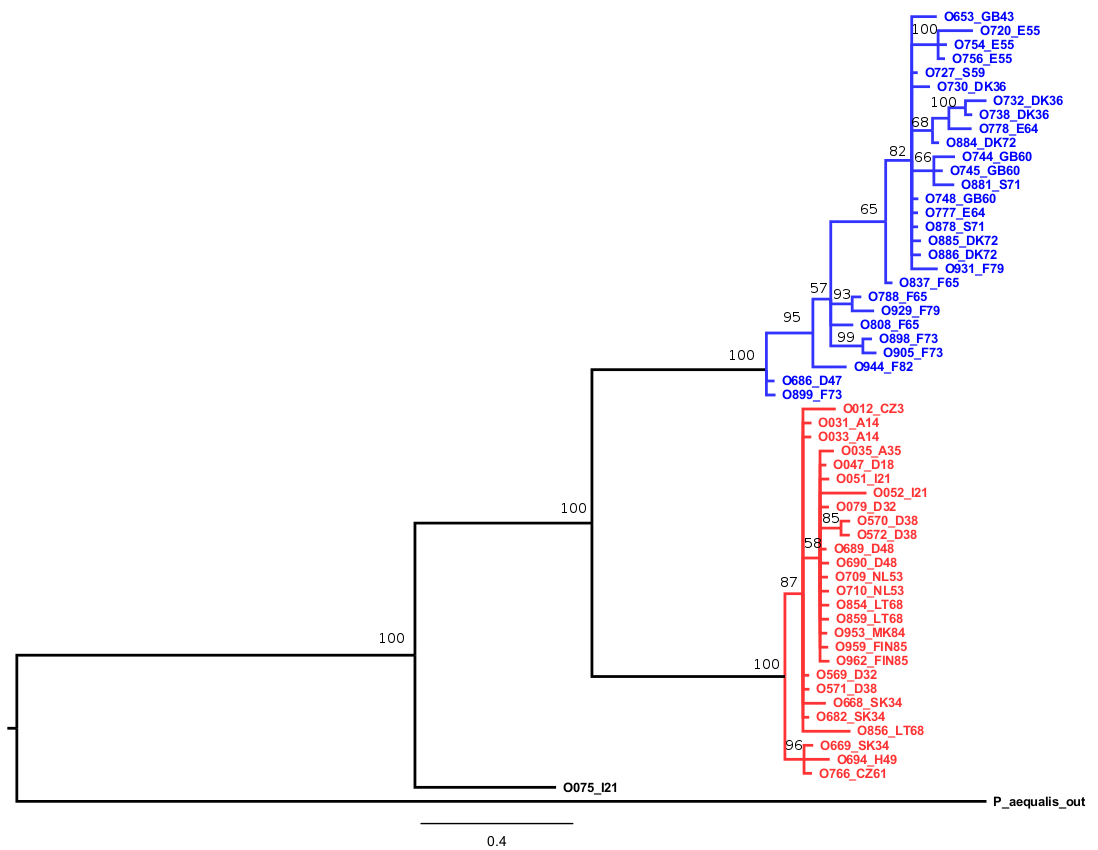

Supplement: Figure S1 — Bayesian 50% majority rule consensus tree of COI and ITS 2 sequences combined of Orthopelma mediator. The number at each node indicates posterior probability values. With the taxon labels the country and number of sampling site are given, see Fig. 3. The western clade is coloured in blue, the eastern in red. (TIFF) [file pone.0047156.s001.tiff]
