# Supplementary material for: No Concordant Phylogeographies of the Rose Gall Wasp Diplolepis rosae (Hymenoptera, Cynipidae) and Two Associated Parasitoids across Europe
Source: PLoS One. 2012 Oct 11;7(10):e47156. doi: 10.1371/journal.pone.0047156 (PMC3469489; doi:10.1371/journal.pone.0047156)
Supplement: Table S1 — Sampling sites where rose galls were collected, collectors, number of individual wasps collected and their haplotype: the gall wasp Diplolepis rosae and its most frequent parasitoids Orthopelma mediator and Glyphomerus stigma. (DOC) [file pone.0047156.s002.doc]

Table S1. Sampling sites where rose galls were collected, collectors, number of individual wasps collected and their haplotype: the gall wasp *Diplolepis rosae* and its most frequent parasitoids *Orthopelma mediator* and *Glyphomerus stigma*.

| Sampling site | | | *D. rosae* | | *O. mediator* | | | *G. stigma* | | Collector |
| --- | --- | --- | --- | --- | --- | --- | --- | --- | --- | --- |
| Location | Long. | Lat. | Ind. | *COI* | Ind. | *COI* | *ITS* 2 | Ind. | *COI* |  |
| **Austria** |  |  |  |  |  |  |  |  |  |  |
| Sandeck | 16.80 | 47.76 | 3 | Diplo 3 | 3 | Ortho1 | Hap2 | 1 | Glypho1 | R. Brandl |
| Klagenfurt | 14.33 | 46.63 | 1 | Diplo 2 | 0 |  |  | 0 |  | H. Riegler-Hager; H.J. Wagner |
| Koralpe | 15.12 | 46.82 | 1 | Diplo 1 | 0 |  |  | 0 |  | M. Brändle |
| **Czech Republic** |  |  |  |  |  |  |  |  |  |  |
| Průhonice | 14.55 | 50.00 | 1 | Diplo 1 | 1 | Ortho 1 | Hap1 | 0 |  | Z. Kiesenbauer |
| Olomouc | 17.25 | 49.59 | 2 | Diplo 1 | 1 | Ortho 7 | Hap3 | 1 | Glypho 16 | E. Křístková |
| **Denmark** |  |  |  |  |  |  |  |  |  |  |
| Tversted | 10.18 | 57.58 | 3 | Diplo 8 | 3 | Ortho 12; 13; 14 | Hap7 | 0 |  | H. Adsersen |
| Horsholm | 12.50 | 55.88 | 3 | Diplo 1 | 3 | Ortho 11 | Hap7 | 0 |  | H. P. Ravn |
| **Finland** |  |  |  |  |  |  |  |  |  |  |
| Örö | 22.32 | 59.81 | 3 | Diplo 1 | 2 | Ortho 2 | Hap6 | 0 |  | A. Albrecht |
| **France** |  |  |  |  |  |  |  |  |  |  |
| Nantes | -1.55 | 47.21 | 3 | Diplo 13 | 3 | Ortho 18; 19 | Hap5 | 2 | Glypho 19 | O. Plantard |
| Chelles | 2.59 | 48.88 | 3 | Diplo 7 | 3 | Ortho 8; 20 | Hap4 | 0 |  | A. Kohnen |
| Sarlat-et-Caneda | 1.21 | 44.88 | 3 | Diplo 1 | 2 | Ortho 21; 22 | Hap5 | 0 |  | A. Kohnen |
| Chateau-Challon | 5.61 | 46.75 | 3 | Diplo 1; 4; 7 | 1 | Ortho 23 | Hap8 | 0 |  | A. Kohnen |
| Perigueux | 0.73 | 45.20 | 2 | Diplo 1 | 0 |  |  | 0 |  | A. Kohnen |
| Doucier | 5.55 | 46.18 | 0 |  | 0 |  |  | 2 | Glypho 20; 21 | A. Kohnen |
| **Germany** |  |  |  |  |  |  |  |  |  |  |
| Ulm | 9.96 | 48.42 | 3 | Diplo 4 | 1 | Ortho 2 | Hap2 | 0 |  | P. Zindl |
| Darmstadt | 8.67 | 49.87 | 2 | Diplo 7 | 2 | Ortho 1; 2 | Hap3 | 0 |  | A. Kohnen |
| Salzgitter | 10.39 | 52.11 | 3 | Diplo 1; 4 | 3 | Ortho 1; 2 | Hap3 | 3 | Glypho 5; 6 | A. Marten |
| Sellin | 13.69 | 54.37 | 1 | Diplo 1 | 1 | Ortho 8 | Hap3 | 0 |  | H. Buhr |
| Poel | 11.41 | 53.98 | 1 | Diplo 1 | 2 | Ortho 2 | Hap3 | 0 |  | H. Buhr |
| **Hungary** |  |  |  |  |  |  |  |  |  |  |
| Matrafured | 19.97 | 47.82 | 1 | Diplo 4 | 1 | Ortho 9 | Hap6 | 3 | Glypho 8; 10; 11 | C. Penny |
| **Italy** |  |  |  |  |  |  |  |  |  |  |
| Monte Baldo | 10.80 | 45.78 | 2 | Diplo 1 | 2 | Ortho 2; 3 | Hap3 | 0 |  | M. Brändle |
| Siena | 11.31 | 43.31 | 3 | Diplo 5; 6 | 1 | Ortho 4 | Hap4 | 3 | Glypho 2; 3; 4 | P. Castagnini |
| **Lithuania** |  |  |  |  |  |  |  |  |  |  |
| Kaunas | 23.89 | 54.89 | 0 |  | 3 | Ortho 1; 2 |  | 0 |  | P. Ivinskis |
| **Macedonia** |  |  |  |  |  |  |  |  |  |  |
| Ohrid | 20.80 | 41.11 | 2 | Diplo 14; 15 | 1 | Ortho 2 | Hap3 | 2 | Glypho 23; 24 | S. Trajanovski |
| **Netherlands** |  |  |  |  |  |  |  |  |  |  |
| Haaren | 5.21 | 51.60 | 1 | Diplo 1 | 2 | Ortho 2 | Hap6 | 0 |  | J. Wolfs |
| Meeden | 6.88 | 53.11 | 1 | Diplo 9 | 0 |  |  | 0 |  | J. Bijkerk |
| Haren | 6.60 | 53.16 | 1 | Diplo 1 | 0 |  |  | 0 |  | J. Bijkerk |
| Rhenen | 5.56 | 51.95 | 1 | Diplo 1 | 0 |  |  | 0 |  | J. Bijkerk |
| **Norway** |  |  |  |  |  |  |  |  |  |  |
| Lier | 10.27 | 59.74 | 3 | Diplo 1 | 0 |  |  | 0 |  | L. O. Hansen |
| **Poland** |  |  |  |  |  |  |  |  |  |  |
| Baligrod | 22.33 | 49.48 | 1 | Diplo 1 | 0 |  |  | 0 |  | N. Selva |
| **Slovakia** |  |  |  |  |  |  |  |  |  |  |
| Martin | 18.78 | 49.01 | 3 | Diplo 4 | 3 | Ortho 1; 6; 7 | Hap3 | 3 | Glypho 7; 8; 9 | S. Götzfried |
| **Spain** |  |  |  |  |  |  |  |  |  |  |
| Leon | -5.57 | 42.59 | 3 | Diplo 11 | 3 | Ortho 10; 16 | Hap5 | 3 | Glypho 13;14; 15 | V. Castro-González |
| Huesca | -0.40 | 42.14 | 3 | Diplo 12 | 2 | Ortho 11; 17 | Hap5 | 2 | Glypho 17; 18 | J. D. Moreno Rodríguez |
| Burgos | -3.68 | 42.35 | 1 | Diplo 1 | 0 |  |  | 2 | Glypho 12; 13 | A. Kempel |
| **Sweden** |  |  |  |  |  |  |  |  |  |  |
| Stockholm | 18.06 | 59.33 | 1 | Diplo 1 | 1 | Ortho 11 | Hap5 | 0 |  | B. Gruhne |
| Uppsala | 17.64 | 59.85 | 3 | Diplo 1 | 2 | Ortho 11; 12 | Hap5 | 0 |  | J. Nylander |
| **UK** |  |  |  |  |  |  |  |  |  |  |
| L.Hameringham | 0.05 | 53.16 | 3 | Diplo 8 | 1 | Ortho 5 | Hap5 | 0 |  | A. Dale |
| Langford | -2.82 | 51.45 | 2 | Diplo 1; 10 | 3 | Ortho 11; 15; 16 | Hap5 | 0 |  | J. Boyd |
| **Ukraine** |  |  |  |  |  |  |  |  |  |  |
| Kiew | 30.52 | 50.45 | 3 | Diplo 4 | 0 |  |  | 1 | Glypho 22 | T. Parshikova |
